# Genome-wide investigation of an ID cohort reveals de novo 3′UTR variants affecting gene expression

**DOI:** 10.1007/s00439-018-1925-9

**Published:** 2018-08-10

**Authors:** Paolo Devanna, Maartje van de Vorst, Rolph Pfundt, Christian Gilissen, Sonja C. Vernes

**Affiliations:** 1 Neurogenetics of Vocal Communication Group, Max Planck Institute for Psycholinguistics, Wundtlaan 1, 6525 XD Nijmegen, The Netherlands; 2 Department of Human Genetics, Radboud Institute for Molecular Life Sciences, Donders Centre for Neuroscience, Radboud University Medical Center, Nijmegen, The Netherlands; 3 Donders Institute for Brain, Cognition and Behaviour, Montessorilaan 3, 6525 HR Nijmegen, The Netherlands

## Abstract

Intellectual disability (ID) is a severe neurodevelopmental disorder with genetically heterogeneous causes. Large-scale sequencing has led to the identification of many gene-disrupting mutations; however, a substantial proportion of cases lack a molecular diagnosis. As such, there remains much to uncover for a complete understanding of the genetic underpinnings of ID. Genetic variants present in non-coding regions of the genome have been highlighted as potential contributors to neurodevelopmental disorders given their role in regulating gene expression. Nevertheless the functional characterization of non-coding variants remains challenging. We describe the identification and characterization of de novo non-coding variation in 3′UTR regulatory regions within an ID cohort of 50 patients. This cohort was previously screened for structural and coding pathogenic variants via CNV, whole exome and whole genome analysis. We identified 44 high-confidence single nucleotide non-coding variants within the 3′UTR regions of these 50 genomes. Four of these variants were located within predicted miRNA binding sites and were thus hypothesised to have regulatory consequences. Functional testing showed that two of the variants interfered with miRNA-mediated regulation of their target genes, *AMD1* and *FAIM*. Both these variants were found in the same individual and their functional consequences may point to a potential role for such variants in intellectual disability.

**Electronic supplementary material:**

The online version of this article (10.1007/s00439-018-1925-9) contains supplementary material, which is available to authorized users.

## Introduction

Intellectual disability is a genetically heterogeneous disorder, and severe cases occur in about 0.5% of all children (Ropers 2010). Previous studies of ID cohorts have focused on large copy number variants (CNVs), or single nucleotide variants (SNVs) located in protein coding regions, to discover genetic factors contributing to ID. These studies have used a range of techniques including CNV arrays, whole exome sequencing (WES), and whole genome sequencing (WGS), and have dramatically increased the number of genes that contribute to this disorder. However, 38–73% of ID cases remain unexplained (Bowling et al. 2017; Gilissen et al. 2014; Monroe et al. 2016; Rauch et al. 2012; Hamdan et al. 2014).

Variants that affect non-coding regulatory regions of the genome may influence brain development and function via their critical role in regulating gene expression, and have been previously implicated in neurodevelopmental disorders (Wanke et al. 2018). In this study, we hypothesised that de novo variation in 3′UTR regulatory regions may be a possible mechanism through which non-coding mutations contribute to ID. We interrogated WGS data from a previously described cohort of 50 individuals (Gilissen et al. 2014) diagnosed with severe ID (IQ < 50), and their unaffected parents, to identify de novo 3′UTR mutations and investigate their functional consequences.

## Subjects and methods

**Fig. 1:**
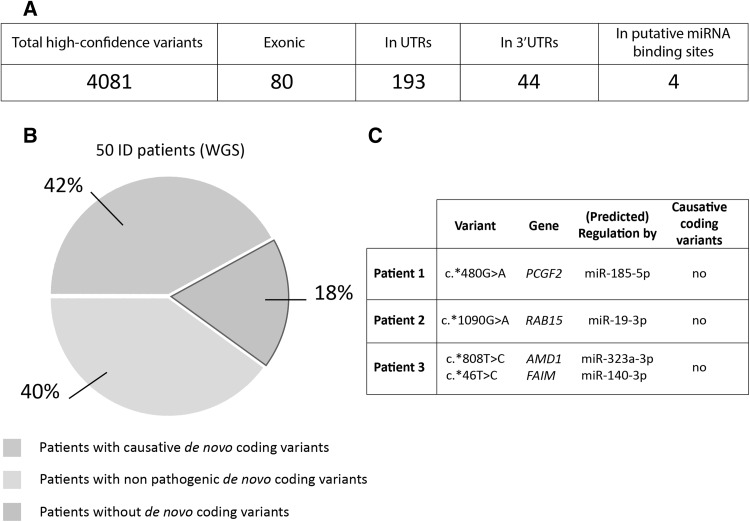
Non-coding variants were identified in a cohort of 50 patients with severe ID. **a** In the cohort of 50 patients, 4081 total, high-confidence de novo variants were identified to be genome wide [refer to Gilissen et al. (2014 ) for the discovery of these variants]. From these, 80 were exonic and 193 were found in UTR regions (5′UTR and 3′UTR). 44 variants were identified in 3′UTRs, of which 4 were predicted to disrupt putative miRNA binding sites. **b** Of the 50 ID patients that were previously sequenced using WGS, 42% of patients received a molecular diagnosis—i.e., de novo coding changes were identified that were thought to be causative. 58% of patients did not carry pathogenic de novo coding mutations; 40% of these had non-pathogenic de novo coding variants, and 18% did not have any de novo changes in coding (exonic) regions (Gilissen et al. 2014). **c** The four de novo variants predicted to disrupt miRNA binding sites were identified in three different patients. Previous studies had not identified any de novo coding mutations in these patients that were likely to explain the phenotype

All 50 patients had a diagnosis of severe intellectual disability (IQ < 50) and had previously been screened via diagnostic genomic CNV arrays, WES and WGS (Gilissen et al. 2014). Within the group of patients that underwent all the diagnostic stages, 21 patients (42%) received a molecular diagnosis as a result of this screening. 20 patients carried coding variants that were not considered pathogenic; the remaining 9 patients did not carry any de novo coding variants [(Gilissen et al. 2014) and see also Fig. 1]. We interrogated the WGS data for this cohort to identify de novo variants that were located within predicted miRNA binding sites in 3′UTR regions. This was performed by overlapping the position of all the de novo variants with the coordinates for miRNA binding sites identified by Targetscan 7.0 (Lewis et al. 2005) using BEDTools (Quinlan and Hall 2010), as described previously (Devanna et al. 2018).

Construct cloning and reporter assays were performed as described previously (Devanna et al. 2018). Briefly, miRNA expression constructs were cloned into pLKO.1 expression vector (Invitrogen) and 3′UTR reporter constructs carrying the reference (+) or variant (Var) sequence were cloned into the pmiR-GLO luciferase expression vector (Promega), using the oligonucleotides described in Table S1. All inserts were confirmed by Sanger sequencing. For functional assays, reporter constructs were co-transfected into HEK293 cells alongside the relevant miRNA expression vector. 48 h post-transfection, firefly luciferase and renilla luciferase activities were measured as per manufacturer’s instructions (Dual Luciferase reporter assay system, Promega). MiRNA sensors (i.e., luciferase reporters designed to be maximally responsive to the cognate miRNA) were included as positive controls to confirm that the overexpressed miRNA was active (see Fig. S1).

**Fig. 2:**
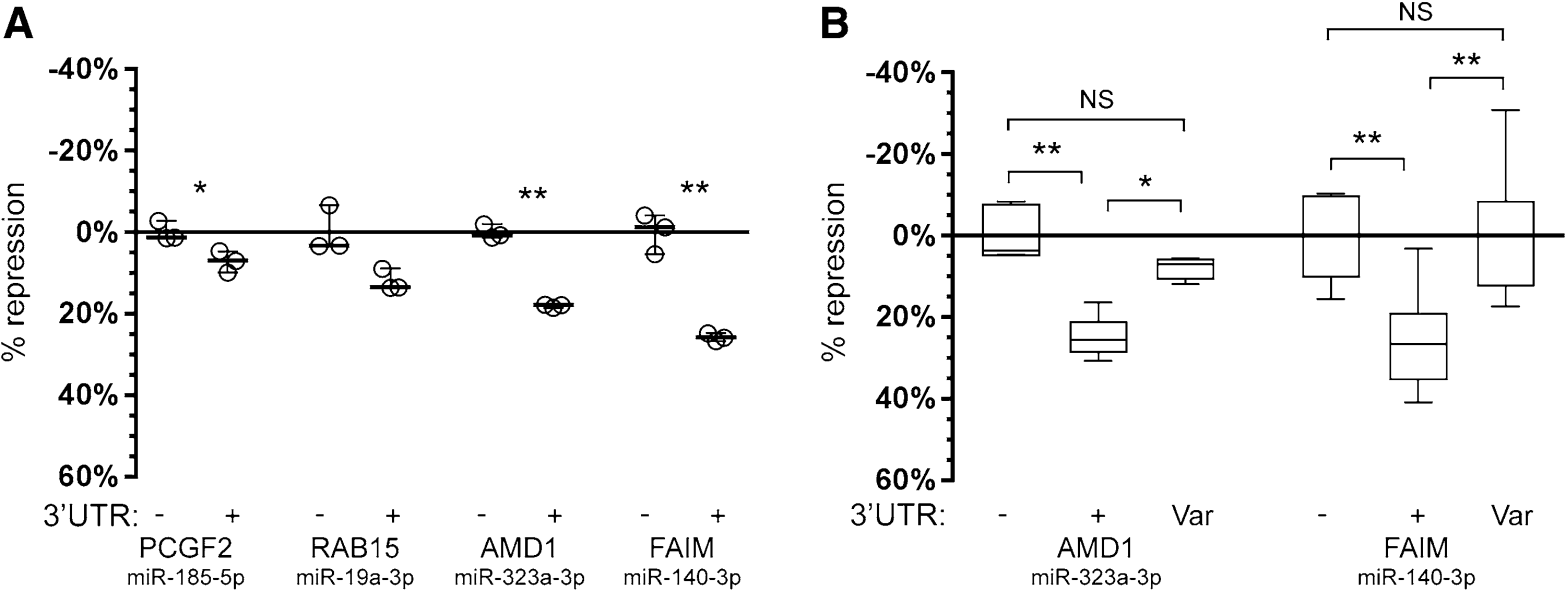
De novo variants identified in an ID cohort disrupt functional miRNA binding sites. **a** Luciferase reporter assays were performed to test the activity of the four predicted miRNA binding sites. Expression of the reporter was strongly (≥ ~ 20%) and significantly (*p* < 0.01) reduced in the presence of miRNA binding sites carrying the reference allele (+) of *AMD1* and *FAIM* compared to an empty vector control (−). Significance was calculated using pairwise *t* test. Results are reported as average of three independent transfections and show the spread of data using the function “min to max” in GraphPad Prism7 (GraphPad Software, La Jolla California USA, http://www.graphpad.com). **b** Introduction of the variant allele identified in the ID patient (“Var”) for both the *AMD1* and *FAIM* binding sites completely abolished repression by the cognate miRNA. Significance was calculated using an ANOVA test, followed by post hoc Tukey calculation. Data are displayed as the percentage of repression observed between the control condition which consists of an empty luciferase reporter with no miRNA binding sites (−) and inclusion of the reference allele (+) or the variant allele (Var). Results are reported as average of three biological replicates (each comprising three independent transfections) and show the spread of data using the function “min to max” in GraphPad Prism7. Statistical significance is indicated as: *p < 0.05; **p < 0.01

Statistical significance was calculated for the reference alleles (Fig. 2a) using a pairwise *t* test, and for the variants (Fig. 2b) via ANOVA followed by post hoc Tukey calculation. All experiments were repeated three times, and within each experiment there were three independent transfections measured for each condition.

## Results

From WGS data of 50 severely affected ID patients, we identified all de novo SNVs located within 3′UTR regions. This gave 44 high-confidence 3′UTR variants. Of these, 4 variants were predicted to disrupt miRNA binding sites. These 4 variants were found in the 3′UTR regions of 4 different genes: *PCGF2* (c.*480G > A), *RAB15* (c.*1090G > A), *AMD1* (c.*808T > C), and *FAIM* (c.*46T > C) (Fig. 1b). Only *PCGF2* has been previously implicated in ID, following the identification of identical missense mutations (NM_007144.2(*PCGF2*):c.194C > T; p.Pro65Leu) in two patients (Deciphering Developmental Disorders Study 2015). Each of these 3′UTR variants were found in patients that, at the time of testing, did not carry any known causal mutation (Fig. 1, Table S2). The 4 variants were predicted to affect the interaction of these 3′UTRs with 4 different miRNAs (miR-185-5p, mir-19a-3p, mir-323a-3p, and mir-140-3p respectively).

Since the 4 miRNA binding sites were identified based on in silico predictions, we first determined if these predicted sites were indeed regulated by miRNAs by performing reporter assays. Constructs carrying the luciferase reporter gene followed by the 3′UTR sequence corresponding to the reference genome miRNA binding site (+) were co-transfected with the relevant miRNAs to determine if the site was regulated (in which case we expect a reduction in reporter gene expression). No reliable change in expression (≥ 20% reduction) was observed for the binding sites in the *PCGF2* and *RAB15* 3′UTR regions (Fig. 2a), suggesting that these predictions were false positives and that they may not represent functional miRNAs sites, at least under these experimental conditions. In contrast, the binding sites identified in the *AMD1* and *FAIM* 3′UTR regions were substantially (≥ 20%) and significantly (*p* < 0.01) down-regulated by their respective miRNAs (mir-323a-3p and mir-140-3p) (Fig. 2a).

Given that the *AMD1* and *FAIM* sites were functionally regulated in the ‘wild type’ reference state (+), we then repeated these experiments, including the identified patient variant (Var) to determine if the presence of the variant would affect this regulation. Indeed, in both cases the presence of the variant abolished repression by each of the relevant miRNAs (Fig. 2b). While the reference allele (+) was again strongly down-regulated, introduction of the variant to the miRNA binding site (Var) led to reporter gene expression that was not significantly different from a control construct (−), which lacked the binding site altogether (Fig. 2b). Interestingly both of the functional variants (within the *AMD1* and *FAIM* 3′UTRs) were found in the same individual. Upon repeated exome sequencing of this patient we have since identified a further de novo mutation in the splice site region of *DNM1* (NM_001288739.1(DNM1):c.1197-8G > A) that is predicted to introduce an alternative splice site resulting in an out-of-frame transcript. Phenotypically this patient presents with IQ < 50, severe hypotonia, severe psychomotor retardation and epilepsy, and congenital thoraco-lumbar scoliosis. These phenotypes match well with previously associated phenotypic features of patients with *DNM1* mutations, thereby making this likely to be the primary cause of disease.

## Discussion

Intellectual disability is a genetically heterogeneous disorder. Genetic screening of large cohorts has implicated > 700 genes in the aetiology of ID (Vissers et al. 2016; Chiurazzi and Pirozzi 2016) reflecting the complexity of the underlying genetic and molecular mechanisms. To date, these screens have identified putatively causative mutations by focusing on large structural variants that affect the coding region of one or more genes, or single base changes in coding regions that disrupt the sequence and therefore function of a protein. Herein we focused on single nucleotide regulatory variants in patients with severe ID. We identified four de novo 3′UTR variants, across three ID patients from the cohort. Two of these variants had functional consequences for miRNA-mediated regulation of expression and these variants were found in the same patient, but in two different neuronally expressed genes (*AMD1* and *FAIM)*. Although during our investigation this patient was diagnosed with a likely pathogenic de novo splice site mutation in *DNM1*, our results show that these de novo 3′UTR mutations affect gene expression, and as such we cannot exclude that these mutations contribute to the patient’s phenotype.

*AMD1* is highly intolerant for loss-of-function variation (ExAC PLi = 0.90) and as such likely to be a haploinsufficient gene. A*MD1* encodes adenosylmethionine decarboxylase 1 (AdoMetDC). AdoMetDC catalyses the decarboxylation of *S*-adenosylmethionine (AdoMet or SAM), which serves as a major donor of methyl groups in numerous reactions that involve DNA methylation (Yordanova et al. 2018). In addition, *AMD1* is essential for embryonic stem cell self-renewal and is translationally down-regulated on differentiation to neural precursor cells (Zhang et al. 2012). *FAIM* encodes a protein that protects against death receptor-triggered apoptosis and regulates B-cell signalling and differentiation. One transcript isoform is ubiquitously expressed and in neurons promotes NGF-induced neurite outgrowth through NF-кB and ERK signalling. Another (longer) isoform is expressed exclusively in tissues of the nervous system and is also involved in neuronal differentiation (Coccia et al. 2017). It is important to note that, unlike deleterious coding mutations which usually reduce the amount of protein present, the variants identified herein interfere with miRNA regulation of these mRNAs, which would result in overexpression of these proteins. The consequences for overexpression of *AMD1* and *FAIM* proteins are currently unknown.

Mutations affecting protein coding regions can completely disrupt the function of a protein—the so-called loss-of-function (LOF) mutations. Conversely, variants found in non-coding regulatory regions are expected to result in more subtle consequences by affecting how much of the protein is present. As such, these regulatory variants would be expected to contribute to disease via a ‘multi-hit’ model in which multiple mutations would contribute to the phenotype. For example, multiple non-coding variants may need to be present in the same individual to produce a phenotype, or non-coding regulatory variants may be a factor in determining the severity of a phenotype when they are present alongside causative coding mutations. In this study we have specifically focused on regulatory variants affecting the 3′UTR and miRNA-mediated regulation, however, these represent only the tip of the iceberg. In addition, future studies should consider the entire breadth of regulatory variants encompassing both 3′UTRs and 5′UTRs, alongside promoters and distal regulatory elements (e.g., enhancers) to determine the genetic bases of ID as this might uncover novel genetic mechanisms that play a role in ID. Looking at the whole spectrum of de novo variants, both in coding and non-coding regulatory regions, will be crucial for a full understanding of the genetic architecture underlying ID.

Our data show that regulatory variation in 3′UTRs can occur de novo in ID patients and have functional effects on gene expression. This new perspective shows the potential of non-coding variants to contribute to ID phenotypes—a category of variation that has so far been overlooked. Although the prevalence of variants disrupting miRNA binding sites remains to be investigated in larger cohorts, we have shown that their biological effects at the level of gene expression make them important factors to consider when trying to disentangle the genetic architecture of intellectual disability.

## Electronic supplementary material

Below is the link to the electronic supplementary material.

Supplementary material 1 (DOCX 86 KB)
