# Supplementary material for: Genome-wide investigation of an ID cohort reveals de novo 3′UTR variants affecting gene expression
Source: Hum Genet. 2018 Aug 10;137(9):717–21. doi: 10.1007/s00439-018-1925-9 (PMC6153495; doi:10.1007/s00439-018-1925-9)
Supplement: Supplementary file 1 — Supplementary material 1 (DOCX 86 KB) [file 439_2018_1925_MOESM1_ESM.docx]

**Supplementary table 1.** Primers and oligonucleotide sequences used for cloning. Restriction site tags

| **3' UTR reporters** | **SENSE OLIGO (5’→3’)** | **ANTISENSE OLIGO (5’→3’)** |
| --- | --- | --- |
| **AMD1** | **TCGAG**AGCCTCTCTTTTATTGTAATGTG**ATATTC**AGCCTCTCTTTTATTGTAATGTG**T** | **CTAGA**CACATTACAATAAAAGAGAGGCT**GAATAT**CACATTACAATAAAAGAGAGGCT**C** |
| **AMD1 (Var)** | **TCGAG**AGCCTCTCTTTTATTGCAATGTG**ATATTC**AGCCTCTCTTTTATTGCAATGTG**T** | **CTAGA**CACATTGCAATAAAAGAGAGGCT**GAATAT**CACATTGCAATAAAAGAGAGGCT**C** |
| **FAIM** | **TCGAG**AGGACTTTTTAATTACTGTGGTA**ATATTC**AGGACTTTTTAATTACTGTGGTA**T** | **CTAGA**TACCACAGTAATTAAAAAGTCCT**GAATAT**TACCACAGTAATTAAAAAGTCCT**C** |
| **FAIM (Var)** | **TCGAG**AGGACTTTTTAATTACTGTGGCA**ATATTC**AGGACTTTTTAATTACTGTGGCA**T** | **CTAGA**TGCCACAGTAATTAAAAAGTCCT**GAATAT**TGCCACAGTAATTAAAAAGTCCT**C** |
| **PCGF2** | **TCGAG**CTTTCTCTCCCCGTTTCTCTCCC**ATATTC**CTTTCTCTCCCCGTTTCTCTCCC**T** | **CTAGA**GGGAGAGAAACGGGGAGAGAAAG**GAATAT**GGGAGAGAAACGGGGAGAGAAAG**C** |
| **PCGF2 (Var)** | **TCGAG**CTTTCTCTCCCCGTTTCTTTCCC**ATATTC**CTTTCTCTCCCCGTTTCTTTCCC**T** | **CTAGA**GGGAAAGAAACGGGGAGAGAAAG**GAATAT**GGGAAAGAAACGGGGAGAGAAAG**C** |
| **RAB15** | **TCGAG**GGCCAGGGACAGCAGTTTGCACA**ATATTC**GGCCAGGGACAGCAGTTTGCACA**T** | **CTAGA**TGTGCAAACTGCTGTCCCTGGCC**GAATAT**TGTGCAAACTGCTGTCCCTGGCC**C** |
| **RAB15 (Var)** | **TCGAG**GGCCAGGGACAGCAGTTTGTACA**ATATTC**GGCCAGGGACAGCAGTTTGTACA**T** | **CTAGA**TGTACAAACTGCTGTCCCTGGCC**GAATAT**TGTACAAACTGCTGTCCCTGGCC**C** |
| **miR-323a-3p sensor** | **TCGAG**AGAGGTCGACCGTGTAATGTG**GAATAT**AGAGGTCGACCGTGTAATGTG**T** | **CTAGA**CACATTACACGGTCGACCTCT**ATATTC**CACATTACACGGTCGACCTCT**C** |
| **miR-140-3p sensor** | **TCGAG**CCGTGGTTCTACCCTGTGGTA**GAATAT**CCGTGGTTCTACCCTGTGGTA**T** | **CTAGA**TACCACAGGGTAGAACCACGG**ATATTC**TACCACAGGGTAGAACCACGG**C** |
| **miR-185-5p sensor** | **TCGAG**TCAGGAACTGCCTTTCTCTCCA**GAATAT**TCAGGAACTGCCTTTCTCTCCA**T** | **CTAGA**TGGAGAGAAAGGCAGTTCCTGA**ATATTC**TGGAGAGAAAGGCAGTTCCTGA**C** |
| **miR-19a-3p sensor** | **TCGAG**TCAGTTTTGCATAGATTTGCACA**GAATAT**TCAGTTTTGCATAGATTTGCACA**T** | **CTAGA**TGTGCAAATCTATGCAAAACTGA**ATATTC**TGTGCAAATCTATGCAAAACTGA**C** |
| **miRNAs** | **FORWARD PRIMER (5’→3’)** | **REVERSE PRIMER (5’→3’)** |
| **miR-323a** | **TTACTACCGGT**CCTGGTATTTGAAGATGCGG | **TTACTGAATTC**CACATCACAAACCCCGCTGGGTC |
| **miR-185** | **TTACTACCGGT**GGCAAAGGCAAGGTCACAGGTCG | **TTACTGAATTC**CCAAGGGAAGGCCATAAACAGATCTC |
| **miR-140** | **TTACTACCGGT**CCCGCTTGGTGTTGGGTTAACTTGC | **TTACTGAATTC**CGCATTCATCTGAACCAACACCCGT |
| **miR-19a** | **TTACTACCGGT**GGTGCATCTAGTGCAGATAGTGAAG | **TTACTGAATTC**CAGAAGCTGTCACATCAGATAGACC |

and spacers between individual miRNA binding sites are highlighted in BOLD, variants are underlined.

| **Patient nr.** | **Identified 3’UTR de novo mutations** | **Associated gene** | **Patient ID in the WGS study^1^** | **Identified de novo mutations in the WGS study** | **Diagnostic interpretation** |
| --- | --- | --- | --- | --- | --- |
| 1 | Chr17(GRCh37): g.36890996G>A | *PCGF2* | 42 | Chr6(GRCh37): g.143092683C>T | Not causative |
| 2 | Chr14(CRCh37): g.65413982G>A | *RAB15* | 38 | Chr1(GRCh37): g.78511993G>T | Not causative |
| 3 | Chr6(GRCh37): g.111215627T>C  Chr3(GRCh37): g.138351971T>C | *AMD1*  *FAIM* | 11 | - |  |

**Supplementary Table 2. Patient identifiers and mutations^1^**

**^1^**Gilissen et al. Nature (2014)


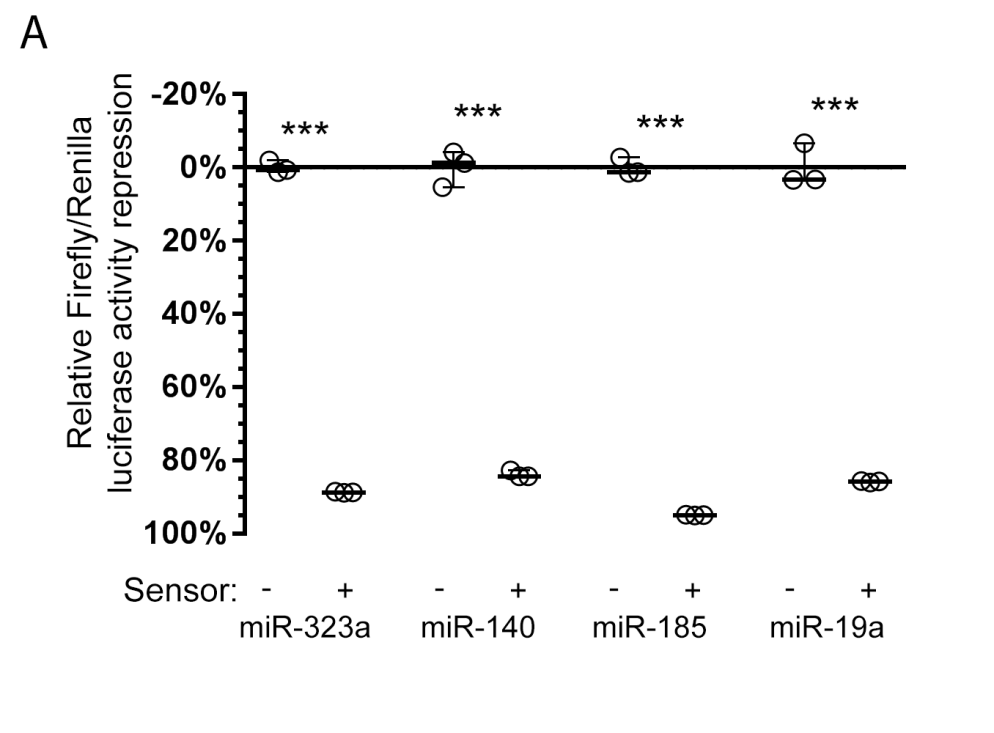


**Supplementary Figure 1.** MiRNA sensors (fully complementary target sequences, designed to be highly responsive to the cognate miRNA) were cloned downstream of the luciferase reporter (as 3'UTRs) to test the expression of miRNAs from the plasmids in experimental conditions (for the sequences see supp. Table 1). Overexpression of the cognate miRNA is expected to strongly repress the expression of the reporter carrying the relative sensor sequence. Repression was detected in all conditions, showing that under experimental conditions the microRNAs were successfully expressed from the plasmids and highly active. Statistical significance was tested using pairwise t-test. *** = p<0.001
